# Patterns and Predictors of Smokeless Tobacco Use among Adults in Bangladesh: Findings from the International Tobacco Control (ITC) Bangladesh Survey

**DOI:** 10.1371/journal.pone.0101934

**Published:** 2014-07-09

**Authors:** Abu S. Abdullah, Pete Driezen, Ummul H. Ruthbah, Nigar Nargis, Anne C. K. Quah, Geoffrey T. Fong

**Affiliations:** 1 Boston University School of Medicine, Boston Medical Center, Boston, Massachusetts, United States of America; 2 School of Public Health, Guangxi Medical University, Nanning, China; 3 Propel Centre for Population Health Impact, University of Waterloo, Waterloo, Ontario, Canada; 4 Department of Economics, University of Dhaka, Dhaka, Bangladesh; 5 World Health Organization, Geneva, Switzerland; 6 Department of Psychology, University of Waterloo, Waterloo, Ontario, Canada; 7 Ontario Institute for Cancer Research, Toronto, Ontario, Canada; The University of Auckland, New Zealand

## Abstract

**Background:**

Although smokeless tobacco (SLT) use is prevalent in South Asian countries including Bangladesh, information about the pattern and correlates of SLT use is scarce. This study described the pattern and predictors of SLT use among Bangladeshi adults.

**Methods:**

The data for this study were derived from the International Tobacco Control Policy Evaluation Bangladesh (ITC BD) Survey, a prospective cohort survey of a nationally representative sample of smokers and non-smokers, conducted during November 2011 and May 2012. The study included 5522 adults aged 15 or above. We used multiple logistic regression models to identify predictors of SLT use.

**Results:**

Of the respondents (N = 5522), 20% were SLT users. In general, SLT use was significantly higher among women, the illiterate and residents of the Dhaka slums or non-tribal/non-border areas outside Dhaka; SLT use increased with age. Several attitudinal factors were also associated with SLT use. Multivariable logistic regression analyses revealed several predictors of SLT use: being female (OR = 1.96, 95% confidence interval, CI: 1.18–3.24), an increasing age, being a resident of a Dhaka slum (OR = 5.86; 95% CI: 3.73–9.21) or non-tribal/non-border areas outside Dhaka (OR = 3.42; 95% CI: 1.94–6.03), being illiterate (OR = 3.37; 95% CI: 1.99–5.71), holding positive opinion towards societal approval of SLT use (OR = 5.84; 95% CI: 3.38–10.09), holding positive opinion towards SLT use by women (OR = 2.63; 95% CI: 1.53–4.54), believing that SLT is addictive (OR = 2.96; 95% CI: 1.51–5.81), and believing SLT is less harmful than bidi (OR = 2.22; 95% CI: 1.36–3.62).

**Conclusion:**

The findings suggest that coordinated efforts of governmental and non-governmental organizations, targeting both smoked tobacco and SLT use reduction and cessation, could be modified to reach each level of population including those who are marginalized, female, less educated and elderly. As most tobacco control programs in Bangladesh target mainly cigarette or bidi smoking, coordinated programs are needed that will also include SLT use within the tobacco control policy and prevention strategies.

## Introduction

Smokeless tobacco (SLT), a general term used in the United States for chewing tobacco and oral snuff products [1] refers to those indigenous smokeless tobacco products that are most frequently used in South Asia, including but not limited to paan, paan masala, zarda, betel quid with tobacco, and gutka [2], [3]. SLT can cause oral cancer [4], [5] and nicotine addiction [6] and is associated with several other health conditions including oral pain [7], cardiovascular diseases [8], hypertension [9], diabetes [10], loss in bone density [11], and problems during pregnancy and following childbirth [12]. Of the estimated 300 million SLT users globally, 27 million are Bangladeshi adults [13]. The World Health Organization (WHO) estimated that 57,000 people die each year from tobacco-attributable (almost all of it in the form of cigarette smoking) diseases in Bangladesh [14]. In a recent study, Alam et al. reported 25% of all deaths in Bangladeshi men aged 25 to 69 years are attributable to cigarette smoking [15]. These mortality rates underestimate total mortality attributable to tobacco because many Bangladeshi men and women use smokeless tobacco [16]. However, these mortality rates provide an estimate of the bigger public health problem associated with tobacco and smokeless tobacco use in Bangladesh – the world’s seventh most populous nation with a population of 150 million. The total cost of SLT-associated diseases in India was US$389 million in 2004 [17], suggesting a high economic burden from SLT use in Bangladesh too. These health and economic consequences underscore the need for urgent public health action to avert the tobacco and SLT-attributable morbidity and mortality in Bangladesh.

Bangladesh has a long history of tobacco use and a variety of ways in which tobacco is smoked and smokeless tobacco is used [18]. The limited data that are available on SLT use in Bangladesh indicate an increasing trend in both male and female adults (aged 15 and above). SLT use has increased from 19.7% (14.8% male and 24.4% female) in 2004 [19] to 27.2% (26.4% male and 27.9% female) in 2009 [16]. By converting these percentages in numbers, we can estimate that the number of SLT users increased by 9 million in five years (from 17.3 million to 25.9 million). Historically, prevalence of smokeless tobacco use is greater among women than among men. However, the growth in SLT use was greater among men during this period.

While these nationally representative prevalence data on SLT use illustrate the magnitude of SLT use in Bangladesh, the socio-demographic, environmental and behavioral level predictors of SLT use are poorly understood. A case-control study of 704 SLT users examined respondents’ perceptions and experiences of SLT use and reported associated factors of SLT use: older age, women, widowed, less educated, housewives, and lower socioeconomic status [20]. However, this study was conducted among hospital patients and in an urban setting, limiting it’s representativeness at the national level. There has been no comprehensive study on the pattern of SLT use, the determinants of usage or on the correlation between SLT use and smoked tobacco in Bangladesh. Given the paucity of such data on SLT use in Bangladesh, this study examined the patterns and predictors of SLT use among Bangladeshi adults by using data from the Wave 3 International Tobacco Control (ITC) Policy Evaluation Bangladesh Project.

## Methods

### Data source

Data analysed in this paper came from the Wave 3 ITC Bangladesh (ITC BD) Survey conducted between November 2011 and May 2012. A detailed description of the ITC BD Survey can be found elsewhere [21]–[23]. Briefly, the ITC BD Survey is a prospective cohort survey of a nationally representative sample of tobacco users and non-users conducted in all six administrative divisions of Bangladesh: Barisal, Chittagong, Dhaka, Khulna, Rajshahi, and Sylhet. The target population of the ITC BD Survey consists of users and non-users of tobacco who are 15 years or older. The Wave 1 survey was conducted in 2009 using a multi-stage probability sampling design. The total sample consisted of two sub-samples: a national sample designed to represent the population of Bangladesh and an urban slum sample, designed to represent the urban poor population of Dhaka. The national sample selected 23 of the 64 districts covering Bangladesh with probability proportional to population size. Another three districts were chosen purposively to include the tribal populations (Garo and Chakma) and a border district between India and Bangladesh. Within randomly selected districts, two upazilas and two villages within upazila were randomly selected, again with probability proportional to population size. A total of 80 villages were selected for the national subsample. Villages contained 300–600 households; a maximum of 450 households per village were enumerated. Households from the enumeration were randomly selected to participate in the Wave 1 survey. The use of a multi-stage probability sampling design guarantees that, in the sense of the theory of probability sampling, the sample is geographically representative of the Bangladesh population.

Household enumeration was conducted to determine basic sociodemographic information of household members as well as tobacco use status of all household members aged 15 or older. Based on the enumeration survey, households were classified into socioeconomic tertiles using a CASHPOR Housing Index. Within each tertile, 10 households having at least one smoker and three households having all non-smokers were randomly chosen to participate in the survey and approached to be interviewed; households that did not respond were replaced. A total of 31,137 households were enumerated in Wave 1, containing 92,853 adults. Of those enumerated, 2000 households were randomly selected for the main survey. One randomly selected adult was interviewed in non-smoking households. In smoker households, all available smokers were selected along with one randomly selected non-smoker.

In the urban slum subsample, 552 households were selected from six urban slums within the city of Dhaka and its surrounding areas. Unlike the national sample, the urban slum sample was designed as a cross-sectional survey due to the insecure nature of housing arrangements in urban slums. In each of the slums, a random starting point was identified and all households in a row were interviewed until the designated number of households for that area had been interviewed. One non-smoker was randomly selected to be interviewed in each household along with all smokers.

Sampling weights were computed for all respondents so that the final sample is broadly representative of the Bangladesh population of adults aged 15 or older. Sampling weights were calibrated to population estimates within predefined geographic and demographic groups (see Wave 1 technical report for more information about the construction of sampling weights).

In Wave 1, 5,771 respondents were interviewed: 1137 were from the urban slums, 256 were from tribal & border areas, 598 were from the city of Dhaka and 3780 were from areas outside the city of Dhaka. Of the 5,771 respondents, 3111 were smokers and 2660 were non-smokers. In Waves 2 and 3, respondents lost to attrition were replaced with newly randomly selected respondents, using the frame of households enumerated in Wave 1. Retention rates were very high from Wave 1 to Wave 2 and from Wave 2 to Wave 3 in the national and purposive samples. Overall, 94% of Wave 1 respondents (not from the urban slums) were re-contacted in Wave 2 while 90% of Wave 2 respondents were re-contacted in Wave 3. None of the Wave 1 slum subsample was followed to Wave 3 so that in Wave 3, 1055 new respondents were recruited to participate in the Wave 3 survey using the same sampling protocol followed in Wave 1 for the slum subsample.

Data were collected using face-to-face interviews. Sampling weights were computed so that results are representative of the Bangladeshi adult population (15 and older).

In all the waves, written consent was obtained from those respondents who can read and write; others gave consent with fingertips. Ethical approval was obtained from the Office of Research Ethics at the University of Waterloo (Waterloo, Canada) (Number. 15019, dated October 03, 2008), and the Ethical Review Committee, Bangladesh Medical Research Council (Number: BMRC/ERC/2007–2010/1372; dated February 19, 2009).

### Measures

A standardized Bengali questionnaire was used for data collection. Details of the questionnaire are described elsewhere [22]. Briefly, the questionnaire obtained information on the subject’s *socio-demographic background* (sex, age, religion, residence (Dhaka non-slum, Dhaka slums, non-tribal/non-border areas outside Dhaka and tribal/border areas), marital status, educational attainment, personal monthly income), whether there are any children (aged 14 or below) at home, *smoking behavior* (non-smoker, exclusively cigarette smokers, exclusively bidi smokers, dual users), *use of any smokeless tobacco (SLT)*, and *any other smokers or SLT users at home*. SLT users were those who answered “yes” to the following question: “Do you currently use any smokeless tobacco products at least once a week?”

*Knowledge of the health consequences of SLT use* was also assessed, along with *attitudes towards SLT use*. To measure *knowledge of the health consequences of SLT use*, respondents were asked: “Based on what you know or believe, does SLT cause…?” Respondents were then read a list of three diseases or conditions (mouth cancer, gum disease, difficulty to open mouth). To measure *attitudes towards SLT use*, respondents were asked whether they believe SLT is addictive (with response categories of “strongly agree, agree, neutral, disagree, strongly disagree”), whether SLT is less harmful than cigarettes or bidi (with response categories of “less harmful, more harmful and no difference”). Respondents were also asked about societal attitudes (with response categories of “approve, neutral, disapprove”), whether SLT is acceptable for women (with response categories of “strongly agree, agree, neutral, disagree, strongly disagree”), and their overall opinion about SLT use (with response categories of “very good, good, neutral, bad, very bad”).

### Analyses

The analysis presented here uses descriptive statistics appropriate for complex survey data to estimate the prevalence of current SLT use among Bangladeshi adults. Differences in the prevalence of SLT use across socio-demographic categories and by attitudes toward SLT use were tested using the Rao-Scott chi square test. A logistic regression model then examined predictors of current SLT use. Initial predictors were selected on the basis of the bivariate associations tested with the Rao-Scott chi square test. A backward elimination procedure was conducted within a logistic regression model to select covariates related to current SLT use, using a p-value for inclusion of 0.05 or below. Variables used in construction of the sampling weights (sex, smoking status, and area of residence) were forced into this model to reduce biases in the other coefficients. The final selected model was refit with a weighted logistic regression model that accounts for the stratified multi-stage survey design. The analysis was conducted using the complex survey routines (PROC SURVEYFREQ and PROC SURVEYLOGISTIC) available in SAS Version 9.3 to account for the stratified multi-stage survey design. The only exception to this was the analysis conducted for the selection of predictors of SLT use, which was run using PROC LOGISTIC. Unless otherwise indicated, all results presented in this paper are weighted. In each case, an unweighted analysis was also conducted; since there were no appreciable differences between the two sets of results, only the weighted results are reported here.

## Results

### Sample characteristics

A total of 5522 respondents were interviewed in the Wave 3 of ITC BD Survey; 72% were recruited in Wave 1 (Table 1). Overall, two-thirds (66.4%) of the sample was from non-tribal, non-border areas outside Dhaka and a greater proportion (59.6%) of the sample was male. Most respondents were married (82.1%), Muslim (83.3%), and had at least one child in their home who was 14 years of age or younger (72.6%). Of the respondents, 36% were non-smokers and 20% were current SLT users (Table 1).

**Table 1:** Characteristics of the ITC Bangladesh Wave 3 sample (unweighted), Bangladesh 2011 (N = 5522).

	Frequency#	%
**Wave of recruitment**		
Wave 1	3954	71.6
Wave 2	206	3.7
Wave 3	1362	24.7
**Sampling area**		
Dhaka non-slum sample	557	10.1
Dhaka slum sample	1055	19.1
Non-tribal, non-border areas outside Dhaka	3666	66.4
Tribal & border areas outside Dhaka	244	4.4
**Sex**		
Male	3293	59.6
Female	2229	40.4
**Age group**		
15–24	1190	21.6
25–39	2033	36.8
40–54	1381	25.0
55+	918	16.6
**Marital status**		
Not married	984	17.9
Married	4519	82.1
**Monthly household income**		
<5000 taka	497	9.0
5,000 to <10,000 taka	1995	36.1
10,000 taka or more	2314	41.9
not reported	716	13.0
**Highest level of education**		
Illiterate	1332	24.2
1 to 8 years	2908	52.9
9 years or more	1258	22.9
**Muslim**		
Non-muslim	919	16.7
Muslim	4570	83.3
**Any children in home < = 14**		
No children/no children < = 14	1505	27.4
At least one child < = 14	3987	72.6
**Any (other) smokers in the home**		
No (other) smokers in the home	2175	39.4
At least one (other) smoker in the home	3347	60.6
**Anyone (else) uses smokeless tobacco in the home**		
No one (else) uses smokeless tobacco	2264	41.0
At least one (other) person uses smokeless tobacco	3258	59.0
**Tobacco use status**		
Exclusively cigarettes	1723	31.2
Exclusively bidi	267	4.8
Dual user*	207	3.7
Mixed user**	297	5.4
Smokeless	781	14.1
Quitter	242	4.4
Nonsmoker	2005	36.3
**Current smokeless user**		
Does not currently use smokeless tobacco	4444	80.5
Current smokeless tobacco user	1078	19.5

*those who use both cigarettes and bidi; **those who use cigarette, bidi and SLT.

# Due to the missing values, the total may not add up to the same in some variables.

### Pattern of SLT use

As shown in Table 2, SLT use was significantly higher in women (23.1%) than men (16.5%) and among those who were illiterate (31.9%) than having 1–8 years (21.5%) or 9 or more years (8.6%) of education. SLT use increased significantly with age: 36% of adults aged 55 and older used smokeless tobacco compared to only 8.5% of adults younger than 25. SLT use was also higher among those who lived in a Dhaka slum (29.9%) and among those living in non-tribal/non-border areas outside of Dhaka (20.3%).

**Table 2:** Smokeless tobacco use by demographic characteristics among Bangladeshi residents (weighted), Bangladesh 2011.

				Rao-Scott ChiSq Test		
	Freq.	%	(95% CI)	ChiSq	df	p value
**Sampling area**						
Dhaka non-slum sample	38/557	10.2	(8.4, 12.2)	42.75	3	<0.001
Dhaka slum sample	304/1055	29.9	(27.1, 32.8)			
Non-tribal, non-borderareas outside Dhaka	701/3666	20.3	(16.7, 24.5)			
Tribal & border areasoutside Dhaka	35/244	9.1	(4.3, 18.4)			
**Sex**						
Male	483/3293	16.5	(13.0, 20.6)	8.87	1	0.003
Female	595/2229	23.1	(18.7, 28.1)			
**Age group**						
15–24	88/1190	8.5	(5.5, 13.1)	66.49	3	<0.001
25–39	335/2033	18.1	(14.0, 23.2)			
40–54	366/1381	23.6	(17.9, 30.4)			
55+	289/918	36.4	(30.3, 42.9)			
**Marital status**						
Not married	164/984	18.3	(12.3, 26.3)	0.37	1	0.543
Married	907/4519	20.2	(17.0, 23.9)			
**Monthly household income**						
<5000 taka	95/497	19.5	(14.0, 26.6)	3.46	3	0.326
5,000 to <10,000 taka	400/1995	21.2	(17.2, 25.7)			
10,000 taka or more	441/2314	20.6	(15.1, 27.4)			
not reported	142/716	13.8	(8.4, 21.9)			
**Highest level of education**						
Illiterate	426/1332	31.9	(27.2, 37.0)	112.58	2	<0.001
1 to 8 years	550/2908	21.5	(17.4, 26.3)			
9 years or more	99/1258	8.6	(6.2, 11.8)			
**Muslim**						
Non-muslim	149/919	14.3	(8.6, 22.8)	2.28	1	0.131
Muslim	923/4570	21.1	(17.2, 25.6)			
**Any children in home < = 14**						
No children/nochildren < = 14	307/1505	18.7	(14.8, 23.3)	0.47	1	0.495
At least one child < = 14	764/3987	20.3	(16.4, 24.8)			
**Any (other) smokers in the home**						
No (other) smokersin the home	468/2175	19.6	(15.1, 25.2)	0.02	1	0.896
At least one (other)smoker in the home	610/3347	19.9	(16.8, 23.5)			
**Anyone (else) uses smokeless tobacco in the home**						
No one (else) uses smokeless tobacco	344/2264	20.6	(15.2, 27.2)	0.20	1	0.652
At least one (other)person uses smokelesstobacco	734/3258	19.2	(16.0, 22.9)			

### Attitudes toward SLT use

As shown in Table 3, Bangladeshis held differing opinions and attitudes towards SLT use. Bangladeshis who believed smokeless tobacco was less harmful than smoked tobacco (cigarette or bidi) were significantly more likely to be current SLT users. Likewise, Bangladeshis who believed that society approves of smokeless tobacco use were significantly more likely to use SLT (40%) than those who believed society disapproves of SLT use (16%). A significantly higher percentage of respondents (43%) who agree that it is acceptable for women to use SLT currently use SLT themselves compared to only 16% of those who do not agree. A significantly higher proportion of those who believed that SLT use is addictive were current SLT users (20%) than those who did not believe so (9%).

**Table 3:** Smokeless tobacco use by attitudes toward smokeless tobacco among Bangladeshi residents (weighted), 2011.

				Rao-Scott ChiSq Test		
	Freq.	%	(95% CI)	ChiSq	df	p value
**Societal attitudes toward smokeless tobacco**						
Society approves	203/490	40.1	(30.3, 50.7)	41.26	2	<0.001
Society disapproves	708/4372	15.9	(12.3, 20.4)			
Neither	77/437	15.2	(11.0, 20.6)			
**It is acceptable for women to use smokeless tobacco**						
Agree/strongly agree	237/535	42.6	(33.2, 52.5)	46.98	1	<0.001
Otherwise	793/4858	16.0	(12.9, 19.7)			
**Smokeless tobacco is** **Addictive**						
Agree/strongly agree	1032/5179	19.8	(16.0, 24.2)	8.56	1	0.003
Otherwise	30/239	8.8	(5.0, 15.1)			
**Smokeless tobacco is less harmful than cigarettes**						
Less harmful	265/731	40.1	(30.2, 50.9)	48.11	2	<0.001
More harmful	128/944	15.0	(9.9, 22.2)			
No difference	549/3322	15.3	(12.1, 19.2)			
**Smokeless tobacco is less harmful than bidi**						
Less harmful	316/845	39.7	(30.4, 49.8)	37.69	2	<0.001
More harmful	104/801	14.1	(7.9, 24.0)			
No difference	518/3326	14.8	(11.4, 19.0)			
**Opinion of smokeless tobacco use**						
Otherwise	32/129	26.3	(18.8, 35.4)	3.18	1	0.074
Bad/very bad	1001/5242	18.7	(15.1, 23.0)			
**Smokeless tobacco causes mouth cancer**						
Otherwise	171/634	26.5	(19.3, 35.3)	3.55	1	0.060
Yes	898/4841	18.4	(14.5, 23.1)			
**Smokeless tobacco causes gum disease**						
Otherwise	184/804	22.2	(15.7, 30.5)	1.32	1	0.251
Yes	879/4665	18.6	(15.0, 22.8)			
**Smokeless tobacco causes difficulty in opening mouth**						
Otherwise	296/1397	22.2	(16.3, 29.5)	1.43	1	0.233
Yes	775/4078	18.2	(14.4, 22.8)			

### Predictors of current SLT use

Table 4 presents the results of a multivariable logistic regression model estimating the association between socio-demographic factors, attitudes and opinions toward SLT and current SLT use. The multivariable model revealed the following predictors of SLT use: being female (OR = 1.96, 95% CI: 1.18–3.24), being aged 25–39 (OR = 2.30, 95% CI: 1.49–3.54) or aged 40–54 (OR = 3.67; 95% CI: 1.76–7.65) or aged 55 and above (OR = 4.25; 95% CI: 2.27–7.95), being a resident of a Dhaka slum (OR = 5.86; 95% CI: 3.73–9.21) or non-tribal/non-border areas outside Dhaka (OR = 3.42; 95% CI: 1.94–6.03), being illiterate (OR = 3.37; 95% CI: 1.99–5.71), holding positive opinion towards societal approval of SLT use (OR = 5.84; 95% CI: 3.38–10.09), holding positive opinion towards SLT use by women (OR = 2.63; 95% CI: 1.53–4.54), believing that SLT is addictive (OR = 2.96; 95% CI: 1.51–5.81), and believing SLT is less harmful than bidi (OR = 2.22; 95% CI: 1.36–3.62). In addition, people who believed that SLT was less harmful had 2.12 times greater odds of using smokeless compared to people who thought smokeless was more harmful than cigarettes (p = 0.014) (omnibus test).

**Table 4:** Odds of being a current smokeless tobacco user (weighted; n = 4702), Bangladesh 2011.

			Wald ChiSq Test		
	Odds Ratio	(95% CI)	ChiSq*	df	p value
**Sex**					
Male	1.00	–	6.79	1	0.009
Female	1.96	(1.18, 3.24)			
**Age**					
15–24	1.00	–	21.95	3	<0.001
25–39	2.30	(1.49, 3.54)			
40–54	3.67	(1.76, 7.65)			
55+	4.25	(2.27, 7.95)			
**Sampling area**					
Dhaka non-slum sample	1.00	–	66.59	3	<0.001
Dhaka slum sample	5.86	(3.73, 9.21)			
Non-tribal, non-border areas outside Dhaka	3.42	(1.94, 6.03)			
Tribal & border areas outside Dhaka	3.11	(0.74, 13.03)			
**Income**					
<5,000 taka	1.00	–	3.38	3	0.337
5,000 to<10,000	0.89	(0.57, 1.38)			
10,000+ taka	1.21	(0.76, 1.95)			
Not stated	0.83	(0.42, 1.65)			
**Highest level of education**					
9 years or more	1.00	–	24.20	2	<0.001
1 to 8 years	2.60	(1.76, 3.84)			
Illiterate	3.37	(1.99, 5.71)			
**Societal attitudes toward smokeless tobacco**					
Neither	1.00	–	79.16	2	<0.001
Society disapproves	1.37	(0.90, 2.07)			
Society approves	5.84	(3.38, 10.09)			
**Acceptable for women to use smokeless tobacco**					
Otherwise	1.00	–	12.15	1	<0.001
Agree/strongly agree	2.63	(1.53, 4.54)			
**Smokeless tobacco is addictive**					
Otherwise	1.00	–	9.99	1	0.002
Agree/strongly agree	2.96	(1.51, 5.81)			
**Smokeless tobacco is less harmful than Cigarettes**					
No difference	1.00	–	7.45	2	0.024
More harmful	0.77	(0.33, 1.82)			
Less harmful	1.63	(0.82, 3.22)			
**Smokeless tobacco is less harmful than bidi**					
No difference	1.00	–	13.18	2	0.001
More harmful	1.24	(0.37, 4.13)			
Less harmful	2.22	(1.36, 3.62)			
**Anyone (else) uses smokeless tobacco in the home**					
No one (else) uses smokeless tobacco	1.00	–	1.39	1	0.239
At least one (other) person uses smokeless tobacco	1.63	(0.72, 3.66)			

*Omnibus test.

## Discussion

This study examined the socio-demographic and attitudinal factors associated with SLT use and found that one in every five Bangladeshi adults use SLT. Our sample is nationally representative as the distribution of respondents by division, age group and sex compares well against census estimates from Bangladesh for 2011 (Table S1) [24]. Our findings have some variations with the two earlier studies [16], [19]. As there are methodological differences across studies, a direct comparison of rates needs to be interpreted cautiously.

Our findings show that the overall prevalence of SLT use (20%) is identical to the WHO 2004 study (19.7%) [19]. However, the prevalence of SLT use in our study is lower than the reported estimates (27.2%) in the GATS 2009 study [16] and another 2009 estimate (29.6%) [25]. These differences might be due to the methodological differences across the studies and the timing when the survey was conducted. Moreover, wave 3 survey was conducted in 2011; during the period when several tobacco control programs were funded by the Bloomberg Global Initiative. However, in both the GATS survey and our survey, we observed an apparent rise compared to the rate of 2004. This increasing trend might be due to the increasing taxation on cigarettes, as people from low socioeconomic status smoke more and are responsive to price [23] and the relatively low cost of SLT products. This might be also due to the lack of public awareness and inappropriate knowledge about the harmful effects of SLT use. Also, currently most anti-tobacco campaigns in Bangladesh focuses on cigarette or bidi smoking without any information about SLT use. This creates an opportunity for SLT being offered as an alternative tobacco product for smokers to use instead of cigarette or bidi. This alternative product would be appealing to many smokers who are dependent on nicotine or those who wanted to quit smoking. In this study, 9% of all smokers who had made a serious quit attempt reportedly used SLT to support their most recent quit attempt (data not shown). Also, the Tobacco Control Act (TCA) 2005 initially did not specify SLT in defining tobacco products. Thus most of the measures taken since 2005 to control tobacco use in Bangladesh focused on tobacco smoking. The Amendment to the TCA in 2013 has made provision for the reference to SLT specifically. The rules for the Amendment are in the process of drafting and yet to reflect the measures targeted to SLT control. The oversight of SLT control in the TCA is one of the many reasons that have contributed to the growth of SLT consumption in Bangladesh.

The increased likelihood of SLT use among the illiterate and less educated Bangladeshis may indicate that similar factors are involved in the initiation of SLT use and smoked tobacco among Bangladeshis [16], [19], [22]. Similar socioeconomic differences in SLT use were also reported in India [26], [27]. In an earlier study, we reported that Bangladeshis with low socioeconomic status were more likely to be exposed to tobacco smoke pollution [22]. It is likely that less educated Bangladeshi adults are less aware of tobacco or SLT-related health hazards and find themselves in conditions which predispose them to use SLT. It is also possible that the limited anti-tobacco educational campaigns available in Bangladesh are not reaching this group of people. Also, the sale of SLT products is not monitored cautiously in Bangladesh making it easier for manufacturer and retail stores to promote the products openly to specific populations. Further studies should explore strategies that are used to promote SLT in Bangladesh to guide the development of policy measures.

In this study, Bangladeshis who believed SLT use was less harmful than smoked tobacco (i.e. bidi) used SLT, suggesting that low perception of risk played a key role in encouraging them to use SLT. In other studies misconceptions about the harmlessness of SLT use have led to increased social acceptability and uptake of SLT products [28], [29]. Future prevention strategies should focus on increasing Bangladeshis’ awareness of the relative harms of all tobacco products including SLT use.

The observation that SLT use increases with age is consistent with previous reports [16], [19], [26]. This increased likelihood of SLT use is related to the social acceptance of SLT use by the older people and a greater appeal of cigarette among the younger generations who may be taking up smoking instead of SLT use. Also, this may be due to increased level of nicotine addiction among the population. Following the addiction theory [30], one might have used SLT in few social occasions hooking him/her to the nicotine to develop dependency; overtime he/she will experience growing craving for nicotine and the need for SLT or other tobacco products. Some of this population might switch to tobacco or other smoked tobacco products and some would continue with SLT use. A good proportion of tobacco smokers or quitters may also supplement their tobacco dependency habit by SLT use. In this study, a small proportion of cigarette (4.4%) or bidi (9.2%) smokers who made a serious quit attempt used SLT to support their efforts. Use of SLT may provide a supplementation or substitution of nicotine intake for cigarette smokers [31] encouraging their continued use; this scenario could be particularly true in situations in which smoking is not socially acceptable, but SLT use is.

This study had several limitations. First, because of the cross-sectional design of the study only associations could be explored without any causal relationship. Second, all data on the use of SLT were based on self-reports collected by using an interviewer-administered structured questionnaire. Due to social stigma associated with tobacco and SLT use, people may under-report SLT use [27]. However, self-reported SLT use among adults is a reliable measure and has high agreement with biochemical assessment [32]. Finally, data were collected by trained interviewers who followed written interviewer guidelines. Any difference between their understanding and explanation of the questions asked could result in bias in information collected. However, such bias was minimized by the periodical observation of interviews by the senior research team members and bi-weekly meetings with the interviewers during which any unusual observation was discussed and resolved. Despite these limitations, our study provides recent information on the use of SLT among Bangladeshi adults, which would support evaluation of tobacco control policies in Bangladesh and development of appropriate intervention measures.

## Conclusion

In conclusion, this study identified several important predictors of SLT use in Bangladesh, including being female, increasing age, being illiterate, residing in the Dhaka slum or non-tribal/non-border areas outside of Dhaka, holding positive opinion towards societal approval of SLT use or SLT use by women, and believing SLT is less harmful than bidi. Findings from this study suggest that coordinated efforts of governmental and non-governmental organizations, targeting both smoked tobacco and SLT use reduction and cessation, could be modified to reach each level of population including those who are marginalized, female, less educated and elderly. As most tobacco control programs target mainly cigarette or bidi smoking, coordinated programs are needed that will also include SLT use within the tobacco control policy and prevention strategies. Future studies, possibly qualitative in nature, would be useful to understand the environmental, cultural and societal factors associated with SLT use, particularly among women and among the residents of a Dhaka slum and non-tribal/non-border areas outside Dhaka. Future research should understand the depth of beliefs about SLT use among the public and identify potential interventions that would be acceptable to them. Given the wide acceptance of SLT use by women in Bangladeshi culture, interventions to raise awareness of the harms of SLT use should target women as a key group. At the same time, national programs should focus on changing social norms by addressing inappropriate attitudes and perceptions of risk towards SLT use among the Bangladeshis. Similar to the effective policies for smoked tobacco, policies such as increasing excise tax and restricting marketing of SLT to targeted population including minors, should also be initiated. These interventions should complement the existing intervention strategies aimed at reducing SLT use among the public in Bangladesh.

## Supporting Information

Table S1 **Distribution of ITC Bangladesh Wave 3 respondents by division, age group and sex compared to the 2011 Bangladesh Census.**
(DOC)
