# Supplementary material for: Patterns and Predictors of Smokeless Tobacco Use among Adults in Bangladesh: Findings from the International Tobacco Control (ITC) Bangladesh Survey
Source: PLoS One. 2014 Jul 9;9(7):e101934. doi: 10.1371/journal.pone.0101934 (PMC4090160; doi:10.1371/journal.pone.0101934)
Supplement: Table S1 — Distribution of ITC Bangladesh Wave 3 respondents by division, age group and sex compared to the 2011 Bangladesh Census. (DOC) [file pone.0101934.s001.doc]

**Table S1:** Distribution of ITC Bangladesh Wave 3 respondents by division, age group and sex compared to the 2011 Bangladesh Census.

|  |  |  |  |  |  |  |  |  |  |
| --- | --- | --- | --- | --- | --- | --- | --- | --- | --- |
|  | ***Male*** | | | ***Female*** | | | ***Overall*** | | |
|  | **ITC Bangladesh** | | **2011*** | **ITC Bangladesh** | | **2011*** | **ITC Bangladesh** | | **2011*** |
|  | **Unweighted** | | **Census** | **Unweighted** | | **Census** | **Unweighted** | | **Census** |
| **Age group** | **(n)** | **%** | **%** | **(n)** | **%** | **%** | **(n)** | **%** | **%** |
| **Barisal** | | | | | | | | | |
| 15-24 | (77) | 25.7 | 26.3 | (38) | 21.8 | 25.5 | (115) | 24.3 | 25.9 |
| 25-39 | (97) | 32.3 | 31.0 | (69) | 39.7 | 35.8 | (166) | 35.0 | 33.4 |
| 40-54 | (56) | 18.7 | 23.3 | (46) | 26.4 | 21.1 | (102) | 21.5 | 22.2 |
| 55+ | (70) | 23.3 | 19.4 | (21) | 12.1 | 17.5 | (91) | 19.2 | 18.5 |
| **Chittagong** | | | | | | | | | |
| 15-24 | (122) | 22.2 | 30.5 | (87) | 24.9 | 30.3 | (209) | 23.3 | 30.4 |
| 25-39 | (183) | 33.3 | 31.2 | (132) | 37.8 | 35.1 | (315) | 35.1 | 33.2 |
| 40-54 | (136) | 24.8 | 21.2 | (89) | 25.5 | 20.1 | (225) | 25.1 | 20.6 |
| 55+ | (108) | 19.7 | 17.1 | (41) | 11.7 | 14.6 | (149) | 16.6 | 15.8 |
| **Dhaka** | | | | | | | | | |
| 15-24 | (253) | 17.9 | 25.8 | (249) | 22.9 | 28.2 | (502) | 20.1 | 27.0 |
| 25-39 | (518) | 36.7 | 34.5 | (449) | 41.3 | 37.4 | (967) | 38.7 | 36.0 |
| 40-54 | (381) | 27.0 | 23.6 | (268) | 24.6 | 20.3 | (649) | 26.0 | 21.9 |
| 55+ | (258) | 18.3 | 16.0 | (122) | 11.2 | 14.1 | (380) | 15.2 | 15.0 |
| **Khulna** | | | | | | | | | |
| 15-24 | (64) | 18.6 | 24.9 | (52) | 27.1 | 24.8 | (116) | 21.6 | 24.9 |
| 25-39 | (113) | 32.8 | 32.8 | (63) | 32.8 | 37.6 | (176) | 32.8 | 35.2 |
| 40-54 | (85) | 24.6 | 25.2 | (51) | 26.6 | 21.9 | (136) | 25.3 | 23.6 |
| 55+ | (83) | 24.1 | 17.0 | (26) | 13.5 | 15.7 | (109) | 20.3 | 16.3 |
| **Rajshahi** | | | | | | | | | |
| 15-24 | (115) | 21.1 | 25.2 | (76) | 22.4 | 25.4 | (191) | 21.6 | 25.3 |
| 25-39 | (199) | 36.5 | 34.7 | (133) | 39.1 | 37.9 | (332) | 37.5 | 36.3 |
| 40-54 | (140) | 25.7 | 24.4 | (86) | 25.3 | 21.5 | (226) | 25.5 | 23.0 |
| 55+ | (91) | 16.7 | 15.6 | (45) | 13.2 | 15.2 | (136) | 15.4 | 15.4 |
| **Sylhet** | | | | | | | | | |
| 15-24 | (32) | 22.2 | 29.5 | (25) | 29.1 | 28.6 | (57) | 24.8 | 29.0 |
| 25-39 | (45) | 31.3 | 31.2 | (32) | 37.2 | 35.3 | (77) | 33.5 | 33.3 |
| 40-54 | (32) | 22.2 | 22.6 | (11) | 12.8 | 21.2 | (43) | 18.7 | 21.9 |
| 55+ | (35) | 24.3 | 16.7 | (18) | 20.9 | 14.9 | (53) | 23.0 | 15.7 |
| **All Divisions** | | | | | | | | | |
| 15-24 | (663) | 20.1 | 26.7 | (527) | 23.6 | 27.4 | (1190) | 21.6 | 27.0 |
| 25-39 | (1155) | 35.1 | 33.3 | (878) | 39.4 | 36.9 | (2033) | 36.8 | 35.1 |
| 40-54 | (830) | 25.2 | 23.5 | (551) | 24.7 | 20.8 | (1381) | 25.0 | 22.1 |
| 55+ | (645) | 19.6 | 16.5 | (273) | 12.2 | 14.9 | (918) | 16.6 | 15.7 |
|  |  |  |  |  |  |  |  |  |  |
